# Development and Validation of a CT-Based Radiomics Nomogram for Predicting Postoperative Progression-Free Survival in Stage I–III Renal Cell Carcinoma

**DOI:** 10.3389/fonc.2021.742547

**Published:** 2022-01-27

**Authors:** Haijie Zhang, Fu Yin, Menglin Chen, Liyang Yang, Anqi Qi, Weiwei Cui, Shanshan Yang, Ge Wen

**Affiliations:** ^1^Department of Imaging, Nanfang Hospital, Southern Medical University, Guangzhou, China; ^2^PET/CT Center, Department of Nuclear Medicine, First Affiliated Hospital of Shenzhen University, Shenzhen Second People’s Hospital, Shenzhen, China; ^3^College of Information Engineering, Shenzhen University, Shenzhen, China

**Keywords:** renal cell carcinoma (RCC), Radiomics, CT, progression-free survival (PFS), predict model, artificial intelligence

## Abstract

**Background:**

Many patients experience recurrence of renal cell carcinoma (RCC) after radical and partial nephrectomy. Radiomics nomogram is a newly used noninvasive tool that could predict tumor phenotypes.

**Objective:**

To investigate Radiomics Features (RFs) associated with progression-free survival (PFS) of RCC, assessing its incremental value over clinical factors, and to develop a visual nomogram in order to provide reference for individualized treatment.

**Methods:**

The RFs and clinicopathological data of 175 patients (125 in the training set and 50 in the validation set) with clear cell RCC (ccRCC) were retrospectively analyzed. In the training set, RFs were extracted from multiphase enhanced CT tumor volume and selected using the stability LASSO feature selection algorithm. A radiomics nomogram final model was developed that incorporated the RFs weighted sum and selected clinical predictors based on the multivariate Cox proportional hazard regression. The performances of a clinical variables-only model, RFs-only model, and the final model were compared by receiver operator characteristic (ROC) analysis and DeLong test. Nomogram performance was determined and validated with respect to its discrimination, calibration, reclassification, and clinical usefulness.

**Results:**

The radiomics nomogram included age, clinical stage, KPS score, and RFs weighted sum, which consisted of 6 selected RFs. The final model showed good discrimination, with a C-index of 0.836 and 0.706 in training and validation, and good calibration. In the training set, the C-index of the final model was significantly larger than the clinical-only model (DeLong test, *p* = 0.008). From the clinical variables-only model to the final model, the reclassification of net reclassification improvement was 18.03%, and the integrated discrimination improvement was 19.08%. Decision curve analysis demonstrated the clinical usefulness of the radiomics nomogram.

**Conclusion:**

The CT-based RF is an improvement factor for clinical variables-only model. The radiomics nomogram provides individualized risk assessment of postoperative PFS for patients with RCC.

## Introduction

Renal cell carcinoma (RCC) is a malignant tumor originating from the proximal tubular epithelial system of renal parenchyma, and accounts for about 85% of all adult renal malignant tumors. It is estimated that, in 2020, there were 431,288 new cases of RCC worldwide, resulting in 179,368 deaths, and accounting for 2.2% and 1.8% of global new cancer morbidity and mortality, respectively. In addition, the incidence of renal cancer is increasing yearly (1). The clear cell renal cell carcinoma (ccRCC) is the most common subtype accounting for about 75% of all RCC (2), and is associated with high invasion and poor prognosis (3, 4). According to the AJCC Tumor Classification Criteria eighth Edition (2017) (5), surgery is the preferred treatment for patients with stage I–III RCC, and is associated with a 5-year survival rate of 71% to 91% (6). However, approximately 20% to 30% of patients will relapse after surgery (7). If we can predict these patients with high risk of recurrence before surgery, and give them targeted treatment and close follow-up, it will be very helpful to improve the prognosis of these patients.

Traditional radiotherapy and chemotherapy have poor efficacy for RCC, and there are no effective adjuvant therapies for RCC. A recent clinical trial showed that a subset of patients with more aggressive disease could benefit from targeted therapy after surgery (8). According to National Comprehensive Cancer Network and European Association of Urology guidelines, adjuvant therapy can reduce the recurrence rate of stage III ccRCC, which is associated with a high recurrence risk. However, about 50% of patients in this high-risk subgroup still do not have postoperative recurrence, and do not need to receive expensive adjuvant targeted therapy. Therefore, there is a need to develop prognostic factors to identify patients who will and will not benefit from adjuvant targeted therapy.

There are currently no markers for a diagnosis of RCC. Tumor stage and pathological nuclear grade are the most important prognostic factors. Nevertheless, distinct outcomes are demonstrated in patients with equivalent tumor-node-metastasis stage and pathological grade; they cannot fully address the issue of individualized treatment for patients with different recurrence risks (9, 10).

The field of artificial intelligence and radiomics has developed rapidly in recent years. Many studies have demonstrated that radiomics can be used to assess the heterogeneity of tumors, thus providing clinicians with more accurate prognostic information to inform treatment decisions (11–13). It has been increasingly reported that radiomics can be used for differentiating benign and malignant renal tumors, as well as discriminating high and low Fuhrman nuclear ccRCC (14, 15). However, to the best of our knowledge, no study has evaluated radiomics for its ability to predict the aggressive potential of ccRCC.

The purpose of this study was to investigate Radiomics Features (RFs) associated with progression-free survival (PFS) of RCC, assessing its incremental value over clinical factors, and to develop a visual nomogram in order to provide reference for individualized treatment and prognosis evaluation of RCC.

## Materials and Methods

### Patients

This retrospective study was approved by the Ethics Committee of Southern Medical University, and because of the retrospective nature of the analysis, the requirement of informed patient consent was waived. The data of patients with RCC who were treated in the Department of Urology, Nanfang Hospital, Southern Medical University, from March 2011 to March 2016 were retrospectively collected. Patients were randomly divided into a training set and validation set in a ratio of approximately 7:3. Clinical, pathological, and surgical data collected included age, sex, symptoms (low back pain, hematuria, emaciation, low-grade fever, cough, abdominal mass, and paraneoplastic syndrome), the interval from diagnosis to treatment, Karnofsky performance status (KPS) score, hemoglobin level, serum calcium level, neutrophil count, platelet count, maximum tumor size, stage, and pathological subtype, WHO/ISUP nuclear grade, overall tumor size, growth pattern, necrosis and calcification, treatment method, and adjuvant treatment. Inclusion criteria for the study were as follows: (1) Histological subtype ccRCC; (2) Clinical stage I–III (stage I–II, T1-2N0M0 and stage III, T1-2N1M0, T3N0-1M0); (3) Received radical nephrectomy or partial nephrectomy; and (4) Complete enhanced computed tomography (CT) imaging data, containing non-contrast phase (NCP), cortico-medullary phase (CMP), nephrographic phase (NP), and excretory phase (EP). Patients with a complete cystic renal tumor, positive tumor margins, and inadequate CT images were excluded.

### CT Parameters

A Siemens 64-slice (Somatom Definition CT scanner; Siemens Medical Solutions Company, Malvern, PA, USA) CT scanners were used. A supine CT scan was performed from the diaphragmatic apex to the lower poles of both kidneys with breath holding using the following parameters: CT tube voltage = 120 kV, tube current = 150–320 mA, layer thickness = 5 mm, layer spacing = 5 mm, field of view = 360 mm, matrix = 512 × 512. After obtaining unenhanced images, Omnipaque (GE Healthcare) was injected into the anterior elbow vein with a high-pressure syringe at a dose of 2 ml/kg and an injection rate of 2.5 ml/s, with a maximum dose of 160 ml. Enhanced CT scanning was started at 25–30 s, 75–80 s, and 180–200 s.

### Image Segmentation

Image segmentation was performed by 2 radiologists with 5 and 8 years, respectively, of abdominal imaging diagnosis experience using ITK-snap software. CT images were obtained from the PACS system, with a window width of 300–400 HU, a window level of 45–65 HU, and a slice thickness of 5 mm. The volume of the tumor was selected as the region of interest (ROI), and the edges were kept about 1 mm away from tumor edges to reduce interference from adjacent tissues (such as fat or normal renal tissue). Based on the threshold, areas with CT values less than −55 HU and greater than 350 HU pixels were filtered out. Intra-group and inter-group correlation coefficients (ICCs) were used to ensure stability and repeatability. First, 40 images were randomly segmented by the 2 physicians to assess reproducibility between groups. A week later, Doctor A repeated the same procedure to assess the reproducibility within the group. The results showed that ICC > 0.80 between groups and within groups; it means that the image segmentation was consistent, and the remaining image segmentation was performed by Dr. A.

### Radiomics Feature Extraction and Selection

RFs were extracted, preprocessed, and filtered from segmented images using the PyRadiomics computing platform. First, the original CT images and 3D segmented images were imported into the platform for loading. Then, the image was preprocessed based on the Simple ITK software package embedded in the platform to ensure that the isotropic voxels of texture feature and shape feature are equidistant from adjacent bodies in all directions. Then, the preprocessed image was filtered based on the platform built-in filter, including PyWavelets and Simple ITK for wavelet filter and logarithmic filter, and NumPy for the remaining filters. Finally, four custom feature extraction methods based on the platform were used for radiomics feature extraction.

**Figure 1:**
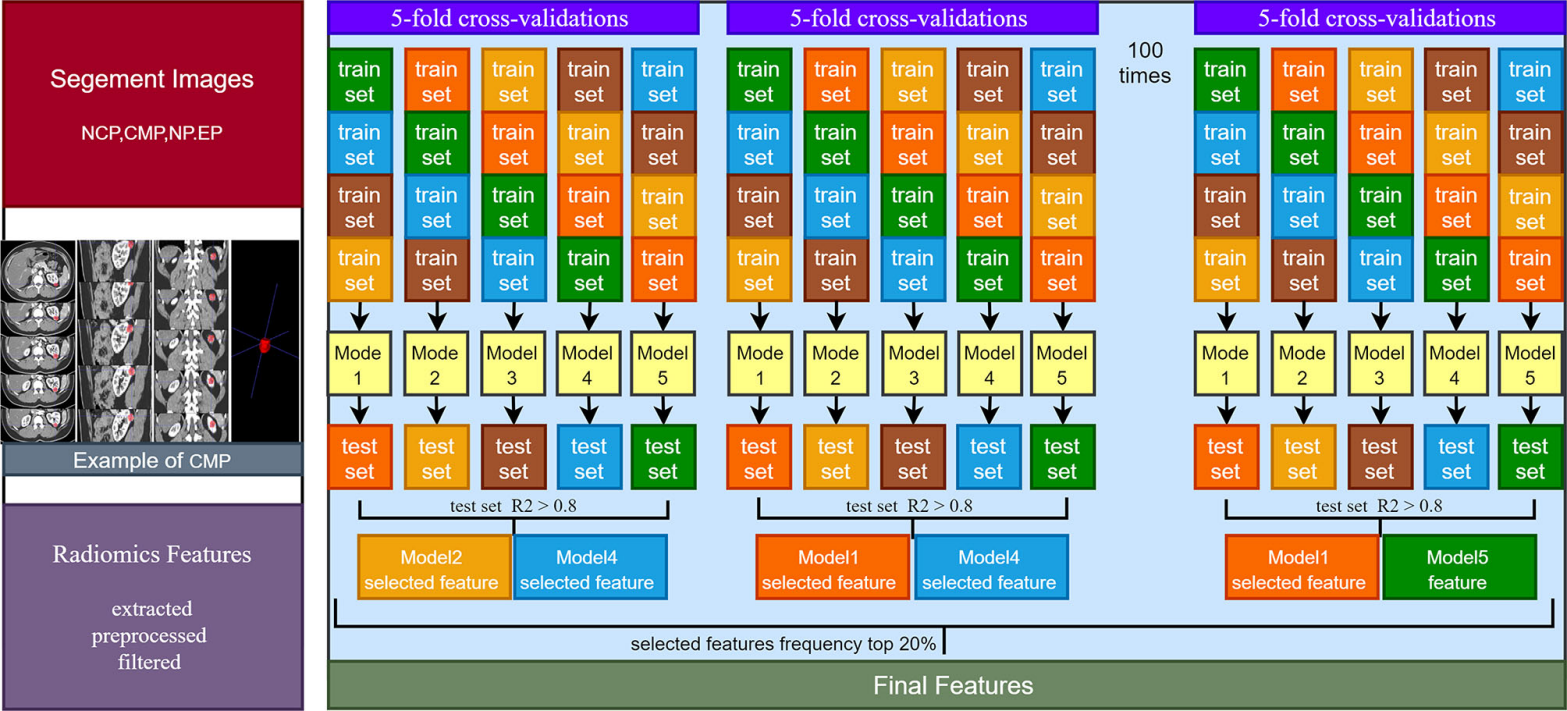
Image segmentation and feature extraction, selection schematic diagram.

In the training set, feature selection was performed based on the stability LASSO algorithm. It performed through 100 times hierarchical 5-fold cross-validation. The LassoCV method used automatically found the optimal penalty coefficient *α* value through k-fold cross-verification. The criterion of *α* selection is to minimize generalization error. RFs were extracted in the training set with 5-fold cross-validations, and RFs with R2 > 0.8 were retained in the 5-fold cross-validation test set. The top 20% RFs of the 100 times 5-fold cross-validation were selected as the final features (**Figure 1**).

### Development of Clinical Variable-Only, RFs-Only, and the Final Model

Multivariate Cox proportional hazard regression models were used to estimate the correlation coefficients of selected RFs to PFS, and to calculate the RFs-weighted sum as an independent variable in both training and validation sets. In the training set, univariate and multivariate Cox proportional hazard regression models were used to investigate factors associated with PFS. Independent variables with a value of *p* < 0.05 in univariate results were entered into a multivariate model, and variables that were significant in the multivariate model were considered factors associated with PFS, and estimated hazard ratios (HRs) were calculated. The final associated factors were used to build a multivariate Cox regression model. The optimal cutoff value of continuous variables was determined by the maximally selected rank statistics from the “maxstat” R package and used to predict PFS. Kaplan-Meier function was used to predict PFS for categorical variables.

### Assessment of the Performance of Different Models

The probabilities were used as an independent continuous variable in receiver operating characteristic (ROC) curve analysis, and the C-index (area under curve the ROC curve [AUC]) and likelihood parameters were determined. A cutoff value was determined by maximizing the Youden index and used to predict PFS. ROC analysis was used in the validation set to assess the final model’s diagnostic effectiveness.

To compare the performance of a clinical variable-only model, RFs-only model, and the final model, ROC analysis and the DeLong test were used. The Hosmer–Lemeshow test was used to check the calibration. Decision curve analysis was used to observe the net benefits. Finally, index net reclassification improvement (NRI) and integrated discrimination improvement (IDI) were used to calculate the increment from the clinical variables-only model to the final model.

### Statistical Analysis

Continuous data were presented as mean ± standard deviation, and categorical data were presented as number and percentage (%). For comparisons of means between groups, Student’s independent *t*-test or Mann–Whitney *U* test was used, depending on normality assumption. Categorical data were tested using chi-square test or Fisher’s exact text (if an expected value ≤ 5 was found). In all analysis, a 2-tailed value of *p* < 0.05 was considered to indicate statistical significance. Statistical analyses were performed using IBM SPSS version 25 software (IBM Corporation, Somers, New York). A nomogram was established using the associated factors in the training set with R statistical software (version 3.5.2) and the “rms” package. The surv_cutpoint function in the “surviminer” package finds the best cutoff value for a continuous variable. The “Predict ABEL” package is used to calculate the NRI and IDI. The decision curve analysis was also performed with R software and “rmda” package.

## Results

### Patient Clinical Characteristics and RFs of the Training and Validation Sets

A total of 175 patients were included in the analysis, with 125 in the training set and 50 in the validation set. The mean age of the training set was 52.31 ± 14.51 years, and that of the validation was 52.06 ± 13.19 years. The male:female ratio of the training set was 2.05:1, and that of the validation set was 1.63:1. The mean PFS of the training set was 55.83 ± 22.25 months, and that of the validation set was 61.06 ± 20.29 months. In the training set and validation set, 32.80% and 24.00% of patients experienced disease progression, respectively. Patient characteristics are summarized in **Table 1**, all clinical characteristics, follow-up results, and RFs of the training set and validation set were comparable (all, *p* > 0.05).

**Table 1:** Patient’s clinical characteristics between training set and validation set.

Parameters	Training set (*n* = 125)	Validation set (*n* = 50)	*p*
Age, years	52.31 ± 14.51	52.06 ± 13.19	0.915
Gender			0.513
Male	84 (67.20%)	31 (62.00%)	
Female	41 (32.80%)	19 (38.00%)	
Symptoms			0.811
No	65 (52.00%)	25 (50.00%)	
Yes	60 (48.00%)	25 (50.00%)	
Interval from diagnosis to treatment			1.000
>1 year	115 (92.00%)	46 (92.00%)	
<1 year	10 (8.00%)	4 (8.00%)	
KPS score			0.064
score ≥80	115 (92.00%)	50 (100.00%)	
score <80	10 (8.00%)	0 (0.00%)	
Hemoglobin			0.451
≥120 g/L	96 (76.80%)	41 (82.00%)	
<120 g/L	29 (23.20%)	9 (18.00%)	
Serum calcium			1.000
<10.2 mg/dl	117 (93.60%)	47 (94.00%)	
>10.2 mg/dl	8 (6.40%)	3 (6.00%)	
Neutrophils			0.367
≤7 × 10^9^/L	109 (87.20%)	46 (92.00%)	
>7 × 10^9^/L	16 (12.80%)	4 (8.00%)	
Platelet			0.240
≤ Normal level	92 (73.60%)	41 (82.00%)	
> Normal level	33 (26.40%)	9 (18.00%)	
Tumor size group			0.737
<40	54 (43.20%)	18 (36.00%)	
40–<70	49 (39.20%)	22 (44.00%)	
70–<100	15 (12.00%)	8 (16.00%)	
≥100	7 (5.60%)	2 (4.00%)	
T stage			0.539
T1	97 (77.60%)	38 (76.00%)	
T2	16 (12.80%)	9 (18.00%)	
T3	12 (9.60%)	3 (6.00%)	
N stage			0.760
N0	115 (92.00%)	47 (94.00%)	
N1	10 (8.00%)	3 (6.00%)	
Clinical stage			0.306
Stage I–I: T_1-2_N_0_M_0_	105 (84.00%)	45 (90.00%)	
Stage III: T_1-2_N_1_M_0_, T_3_N_0-1_M_0_	20 (16.00%)	5 (10.00%)	
WHO/ISUP nuclear grade			0.160
Low	91 (72.80%)	31 (62.00%)	
High	34 (27.20%)	19 (38.00%)	
Grow pattern			0.241
Outside	40 (32.00%)	20 (40.00%)	
Middle	60 (48.00%)	17 (34.00%)	
Inside	25 (20.00%)	13 (26.00%)	
Necrosis			0.956
No	32 (25.60%)	13 (26.00%)	
Yes	93 (74.40%)	37 (74.00%)	
Calcification			0.855
No	101 (80.80%)	41 (82.00%)	
Yes	24 (19.20%)	9 (18.00%)	
Surgery type			0.586
Partial nephrectomy	48 (38.40%)	17 (34.00%)	
Radical nephrectomy	77 (61.60%)	33 (66.00%)	
Adjuvant therapy			0.551
No	107 (85.60%)	41 (82.00%)	
Yes	18 (14.40%)	9 (18.00%)	
PFS, month	55.83 ± 22.25	61.06 ± 20.29	0.152
Progression			0.252
No	84 (67.20%)	38 (76.00%)	
Yes	41 (32.80%)	12 (24.00%)	
1-year survival	121 (96.80%)	49 (98.00%)	1.000
3-year survival	107 (85.60%)	47 (94.00%)	0.122
5-year survival	98 (78.40%)	44 (88.00%)	0.142
RFs			
X1	160.39 ± 181.67	134.74 ± 149.50	0.377
X2	54.58 ± 54.19	59.69 ± 71.86	0.609
X3	37.42 ± 17.05	36.21 ± 15.84	0.665
X4	60.72 ± 34.13	59.03 ± 26.54	0.753
X5	0.21 ± 0.09	0.22 ± 0.09	0.739
X6	59.40 ± 41.28	66.25 ± 66.52	0.412
RFs weighted sum	−1.41 ± 1.28	−1.44 ± 1.31	0.900

NCP, Non-contrast phase; CMP, cortico-medullary phase; NP, nephrographic phase; EP, excretory phase.

A total of 107 RFs were extracted from the 3D multiphase CT images of each phase of each patient. The RF features were categorized as follows: (1) first-order statistics, (2) shape-based features, and (3) texture features. Of the 107 RFs, there were18 first-order statistics features, 14 shape-based features, 24 gray-level cooccurrence matrix (GLCM) features, 16 gray-level size zone matrix (GLSZM) features, 16 gray-level run length matrix (GLRLM) features, 14 gray-level dependence matrix (GLDM) features, and 5 neighboring gray-tone difference matrix (NGTDM) features. A total of 428 (4×107) RFs were extracted from the 4 phase CT images. There were 6 RFs final selected, including X1, X2, X3, X4, X5, and X6. **Table 2** shows RF’s designation, phase, abbreviation, classification, and description. A multivariate Cox regression model for PFS of the training set was established to integrate the RFs indices into a single index, the RFs-weighted sum, using the formula: RFs weighted sum = 5.5056 × 10^-5^ × X1 + 0.0013 × X2 + 0.0028 × X3 + 0.0053 × X4 − 10.4363 × X5 + 0.0048 × X6.

**Table 2:** Patient’s selected RF’s designation, phase, abbreviation, classification, and description.

Phase	Designation	Abbreviation	Category	Description
Corticomedullary phase	Size-Zone Non-Uniformity	X1	GLSZM	The variability of size zone volumes in the image, with a lower value indicating more homogeneity in size zone volumes.
Corticomedullary phase	Complexity	X2	NGTDM	An image is considered complex when there are many primitive components in the image, i.e., the image is non-uniform and there are many rapid changes in gray-level intensity.
Corticomedullary phase	Least Axis Length	X3	Shape	This feature yield the smallest axis length of the ROI-enclosing ellipsoid and is calculated using the largest principal component λ least.
Excretion period	Maximum 2D Diameter Row	X4	Shape	It is defined as the largest pairwise Euclidean distance between tumor surface mesh vertices in the column-slice (usually the sagittal) plane.
Non-enhanced phase	Surface Volume Ratio	X5	Shape	A lower value indicates a more compact (sphere-like) shape. This feature is not dimensionless, and is therefore (partly) dependent on the volume of the ROI.
Parenchyma phase	Maximum 2D Diameter Slice	X6	Shape	It is defined as the largest pairwise Euclidean distance between tumor surface mesh vertices in the row-column (generally the axial) plane.

### Predictive Model of PFS Using the Training Set

The univariate and multivariate Cox regression analyses results of the relations of independent variables to PFS in the training set are shown in **Table 3**. Variables significant in univariate results were entered into the multivariate model. Because of the high correlation between T stage and clinical stage (*r* = 0.63, *p* < 0.001), T stage was excluded in the final model. Thus, the final model for PFS was established using age, clinical stage, KPS score, and RFs-weighted sum (**Table 4**).

**Table 3:** Univariate and Multivariate Cox-regression results in training set.

Parameters	Univariate		Multivariate	
	HR (95% CI)	*p*	HR (95% CI)	*p*
Age, years	1.03 (1.00 to 1.05)	**0.032**	1.04 (1.01 to 1.07)	**0.012**
Gender				
Male	ref.	–		
Female	1.44 (0.77 to 2.68)	0.251		
Symptoms				
No	ref.	–		
Yes	1.06 (0.58 to 1.96)	0.842		
Interval from diagnosis to treatment				
>1 year	ref.	–		
<1 year	1.60 (0.57 to 4.49)	0.376		
KPS score				
score ≥80	ref.	–	ref.	–
score <80	3.21 (1.34 to 7.69)	**0.009**	2.80 (0.99 to 7.94)	**0.035**
Hemoglobin				
≥120 g/L	ref.	–		
<120 g/L	1.54 (0.78 to 3.01)	0.210		
Serum calcium				
<10.2 mg/dl	ref.	–		
>10.2 mg/dl	1.79 (0.64 to 5.03)	0.271		
Neutrophils				
≤7 × 10^9^/L	ref.	–	ref.	–
>7 × 10^9^/L	2.27 (1.08 to 4.77)	**0.031**	1.62 (0.65 to 4.02)	0.299
Platelet				
≤ Normal level	ref.	–	ref.	–
> Normal level	1.88 (1.00 to 3.52)	0.049	1.85 (0.76 to 4.52)	0.176
Tumor size group		**<0.001**		0.181
<40	ref.	–	ref.	–
40–<70	4.84 (1.94 to 12.10)	<0.001	1.41 (0.42 to 4.69)	0.576
70–<100	12.73 (4.44 to 36.51)	<0.001	5.48 (0.81 to 36.84)	0.080
≥100	19.98 (5.76 to 69.30)	<0.001	7.76 (0.84 to 71.25)	0.070
T stage		**<0.001**		**0.015**
T1	ref.	–	ref.	–
T2	2.62 (1.16 to 5.90)	0.021	0.29 (0.08 to 1.05)	0.059
T3	5.65 (2.59 to 12.32)	<0.001	0.04 (0.00 to 0.40)	0.006
N stage				
N0	ref.	–	ref.	–
N1	6.05 (2.60 to 14.11)	**<0.001**	0.20 (0.03 to 1.30)	0.091
Clinical stage				
Stage I–II: T_1-2_N_0_M_0_	ref.	–	ref.	–
Stage III: T_1-2_N_1_M_0_, T_3_N_0-1_M_0_	5.90 (3.05 to 11.41)	**<0.001**	66.14 (6.38 to 685.77)	**<0.001**
WHO/ISUP nuclear grade				
Low	ref.	–		
High	1.86 (0.99 to 3.49)	0.052		
Grow pattern		0.345		
Outside	ref.	–		
Middle	1.00 (0.48 to 2.09)	0.992		
Inside	1.68 (0.74 to 3.82)	0.216		
Necrosis				
No	ref.	–		
Yes	1.83 (0.81 to 4.14)	0.145		
Calcification				
No	ref.	–	ref.	–
Yes	2.34 (1.20 to 4.56)	**0.013**	1.98 (0.84 to 4.66)	0.117
Surgery type				
Partial nephrectomy	ref.	–		
Radical nephrectomy	1.35 (0.71 to 2.59)	0.359		
Adjuvant therapy				
No	ref.	–		
Yes	1.16 (0.49 to 2.77)	0.732		
RFs weighted sum	2.72 (1.95 to 3.78)	**<0.001**	1.95 (1.00 to 3.80)	**0.049**

P values less than 0.05 are in bold.

**Table 4:** Final model by training set.

Parameters	HR (95% CI)	*p*
Age, years	1.01 (0.99 to 1.04)	0.221
Clinical stage		
Stage I–II: T_1-2_N_0_M_0_	ref.	–
Stage III: T_1-2_N_1_M_0_, T_3_N_0-1_M_0_	3.79 (1.85 to 7.76)	<0.001
KPS ranking		
Score ≥80	ref.	–
Score <80	3.14 (1.26 to 7.86)	0.014
RFs weighted sum	2.48 (1.75 to 3.53)	<0.001

**Figure 2:**
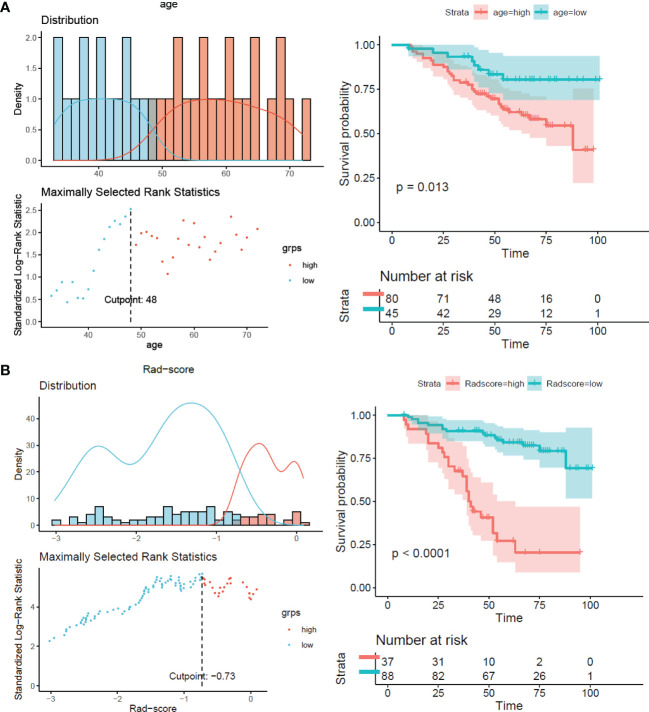
Surv_cutpoint function and survival analysis of PFS in the training set. **(A)** RFs-weighted sum. **(B)** Age.

**Figure 3:**
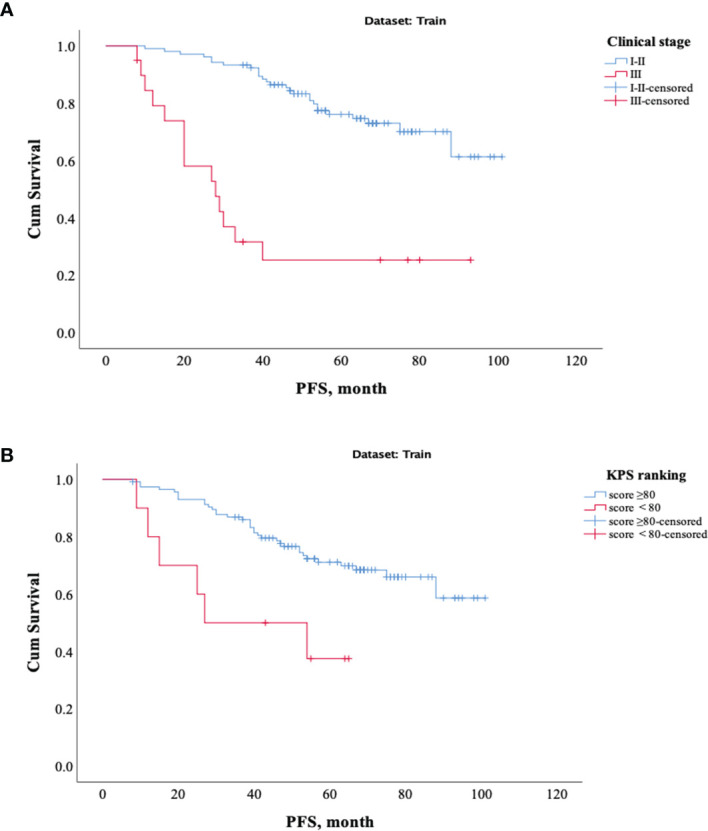
Kaplan–Meier survival analysis of PFS in the training set. **(A)** Clinical stage. **(B)** KPS score.

The results showed that patients with higher age, higher clinical stage, KPS score < 80, and larger RFs-weighted sum were more likely to have disease progression. The surv_cutpoint functions for age and RFs-weighted sum are shown in **Figures 2A, B**, the cutoff value of RFs-weighted sum and age was 48 and −0.73, and significant differences were found between age ≥ 48 and < 48 (*p* = 0.013) and between RFs-weighted sum ≥ −0.73 and < −0.73 (*p* < 0.001).The Kaplan–Meier survival functions for clinical stage and KPS score are shown in **Figures 3A, B**, and significant differences were found between clinical stage I–II and III (*p* < 0.001), and between KPS score ≥ 80 and < 80 (*p* = 0.006).

### ROC Analysis and Nomogram

**Figure 4:**
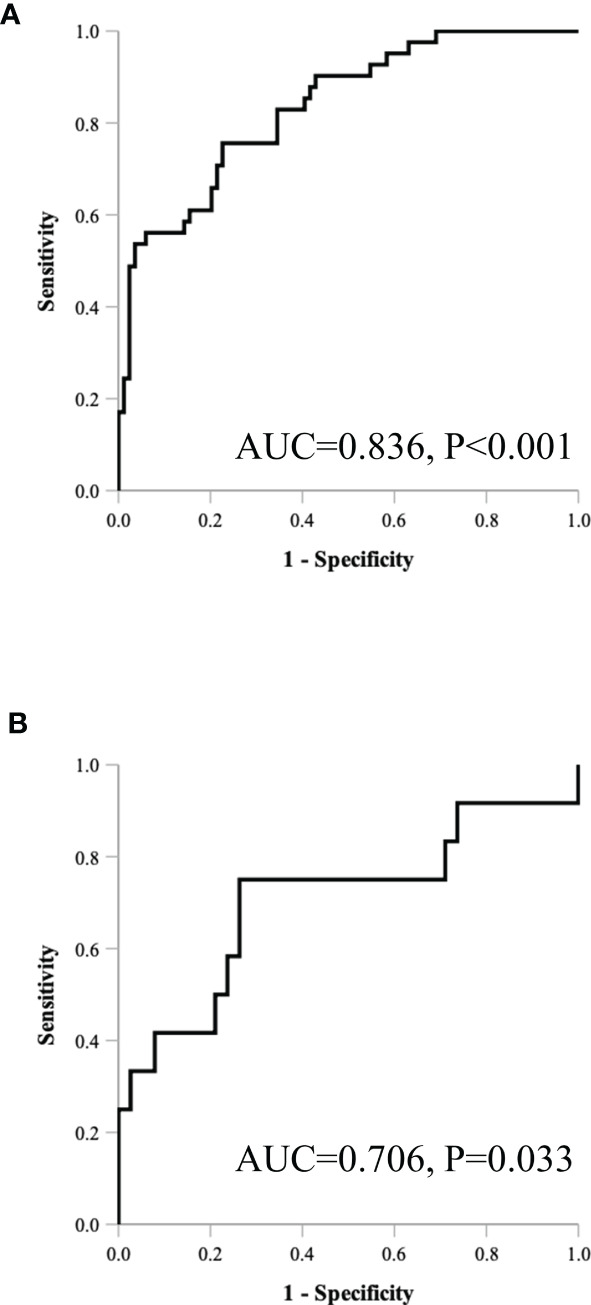
ROC results of the final model of the training set **(A)** and validation set **(B)**.

**Figure 5:**
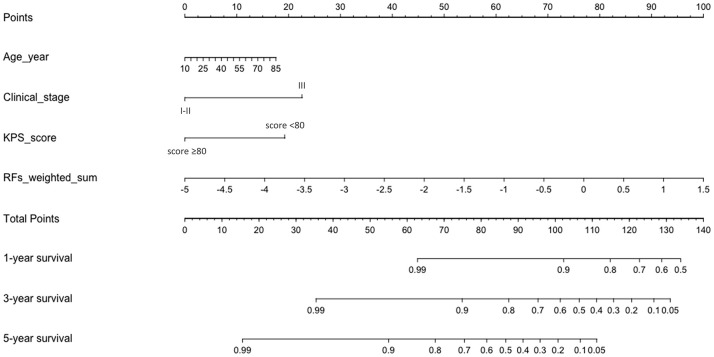
A nomogram for PFS was established that included age, clinical stage, KPS score, and RFs-weighted sum.

**Table 5** and **Figure 4** show the results of ROC analysis of the final models of the training set and validation set. The C-index of training and validation models was 0.836 and 0.706, respectively. The Hosmer–Lemeshow test indicated that the final models of the training set (chi-square = 15.05, *p* = 0.058) and validation set (chi-square = 13.84, *p* = 0.086) were acceptable. A nomogram of the final model was established for clinical use (**Figure 5**), and included risk estimations of PFS, and 1-, 3-, and 5-year survival.

**Table 5:** C-index and diagnostic index of final model in both dataset.

Data set	C-index (95% CI)	*p*	Sensitivity	Specificity	Youden	Accuracy	PPV	NPV	PLR	NLR
Training (*n* = 125)	0.836 (0.763 to 0.909)	<0.001	0.76	0.77	0.53	0.77	0.62	0.87	3.34	0.32
Validation (*n* = 50)	0.706 (0.511 to 0.901)	0.033	0.58	0.74	0.32	0.70	0.41	0.85	2.22	0.57

PPV, Positive predictive value; NPV, negative predictive value; PLR, positive likelihood ratio; NLR, negative likelihood ratio.

### Comparisons of Clinical Variables-Only, RFs-Only, and Final Models

**Figure 6:**
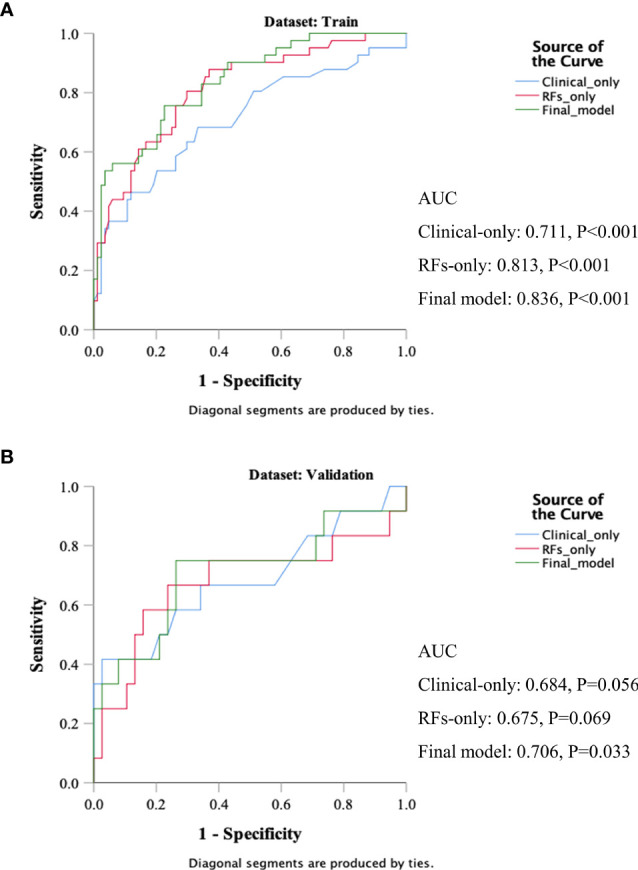
ROC results of the clinical variables-only model, RFs only model, and final model of the training set **(A)** and validation set **(B)**.

Three models were compared to investigate the importance of the RFs indices. The models included a clinical variables-only model (age, clinical stage, and KPS score), RFs-only model (RFs-weighted sum), and final model (age, clinical stage, KPS score, and RFs-weighted sum). The ROC analysis of the models is shown in **Figure 6**. It was found that the C-index of the final model was significantly larger than the clinical-only model (DeLong test, *p* = 0.008), but not significant compared to the RFs-only model (*p* = 0.402). However, no significance was found among the paired comparisons of models in the validation set (all *p* > 0.05).

**Figure 7:**
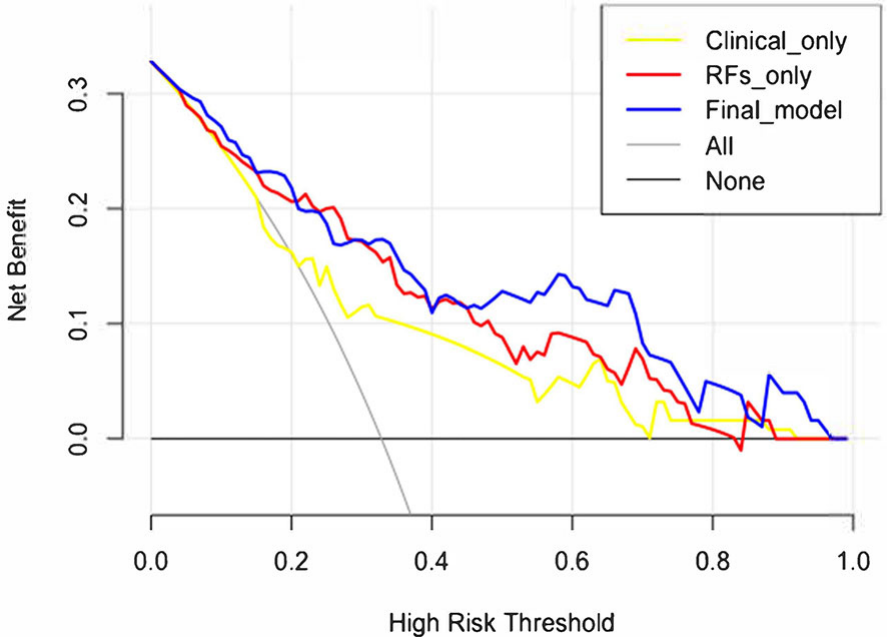
Decision curve analysis results of the clinical variables-only model, RFs only model, and final model.

The decision curve analysis is shown in **Figure 7**. The net benefit was higher in the final model than in the clinical variables-only model all the time and also higher than that of the RFs-only model after a 0.4 risk-threshold. The NRI from the clinical variables-only model to the final model was 18.03% (*Z* = 1.80, *p* = 0.072), marginally significant; and the IDI was 19.08% (*Z* = 6.39, *p* < 0.001).

## Discussion

In this retrospective analysis, we developed a radiomics nomogram that incorporates three clinical factors and RFs-weighted sum for noninvasive, individualized prediction of PFS in patients with clinical stage I–III ccRCC, which can enable physicians to select reasonable treatment tactics and individualized monitoring protocols to improve clinical outcomes. To the best of our knowledge, this is the first prediction model developed to predict PFS of resectable ccRCC using CT-based radiomics. Through cross-validation and calibration, the RFs selection of this study ensures reliability and avoids over-fitting of the model (16). There were 41 progressive cases in the training set used for modeling, and the nomogram contains 4 factors. The variable selection is consistent with the 10–15 EPV (Event per Variable) criteria proposed by Peduzzi et al. (17), indicating that the model is reliable. The proposed radiomics nomogram demonstrated favorable discrimination in both the training set (C-index, 0.836) and validation set (C-index, 0.706), with high sensitivity, specificity, and accuracy.

Many factors, including clinical, anatomical, pathological, and molecular factors and treatment methods, are related to the prognosis of RCC. Early treatment, being asymptomatic, and a higher KPS score (>80) are very important for prolonging the survival of patients with RCC (18). Inflammatory cells (19–21), the TNM classification, and the histological factors included tumor nuclear grade, subtype, sarcomatoid features, microvascular invasion, tumor necrosis, and collection system invasion play a very important role in the tumor prognosis (22, 23). Many molecular factors such as CAIX, VEGF, HIF, Ki67, p53, p21, cell cycle PTEN, E-cadherin, CD44, and CXCR4, as well as other cell cycle and proliferation markers, may be associated with the prognosis of RCC (24–27). Partial nephrectomy is associated with improved survival of early RCC (28). Park et al. (29) reviewed preoperative laboratory data in 747 RCC patients and revealed that clinical information supporting aggressive ccRCC included an older age, larger size, lower hemoglobin, albumin, and calcium, as well as higher platelet and neutrophil. However, few radiologic parameters have been reported as prognostic factors of ccRCC in contrast to pathological markers. We enrolled these variables in this study; univariate Cox regression analysis showed that age, KPS score, neutrophils, tumor size, T stage, N stage, clinical stage, and calcification were associated with PFS, which was consistent with previous studies. However, affected by the RFs weighted sum, the clinical factors of the final model were only age, KPS score, and clinical stage. While RCC can be seen in all age groups, the median age of onset is 64 years, with a high incidence ranging from 50 to 70 years old. The mean age of patients in this study was 52 years, which is lower than that reported in the literature, and this may be related to the small sample size, patient race, and the relatively early disease stage.

Various scoring systems have been developed to predict the risk of postoperative recurrence in patients with RCC. The response evaluation criteria in solid tumors (RECIST) is the most commonly used prognostic evaluation method for tumors (30). However, it is impossible to predict the treatment effect before treatment. Other systems include the University of California, Los Angeles Integrated Staging System (UISS) (31), the Stage Size Grade and Necrosis (SSIGN) model (32), the Leibovich scoring system (33), the Kattan Nomogram (34), and the Karakiewic prognostic model (35). However, some of the parameters used in the model such as tumor necrosis and clinical presentation are subject to inter-observer variability. Different observation end points of different models result in different accuracy results in different studies, and ethnic differences and tumor diversity also limit the use of some systems; in various external validation samples, the results are not consistent (36, 37). Hence, further research and validation are needed.

RFs contain information about tumor heterogeneity and can reflect tumor phenotypes. Our study has filled a gap in the literature on PFS risk of stage I–III RCC in the setting of radiomics. In the recent literature, Radiomics nomogram has demonstrated excellent efficacy in differential diagnosis, nuclear grading, prognosis, and gene expression of RCC (38–43). Among the 6 RFs selected in this study, there were 3 features from the corticomedullary phase, suggesting that the corticomedullary phase may contain more abundant information to predict PFS. The results showed that RFs-weighted sum was an important factor that improved the diagnostic efficiency of the clinical variables-only model. The decision curve analysis revealed that using the radiomics nomogram to predict PFS in patients with stage I–III ccRCC presents more notable benefits than solely relying on clinical variables-only model.

There are several limitations to our study. First, owing to the limitation of the retrospective study and small number of cases, the follow-up time we used was at least 5 years. It would be more interesting to enroll patients without recurrence evidence for more than 5 or 10 years. Second, as a single-center study, the patient population was relatively homogeneous and small. During the 5-year recruiting period, there a large proportion of patients with stage I–II in this study (training set: 84%, verification set: 90%). A large-scale independent prospective multicenter study is needed to evaluate the generalizability of the results, and further work would focus on it.

In conclusion, this study presented a CT-based radiomics nomogram that showed satisfactory performance in predicting PFS in patients with stage I–III ccRCC, as a non-invasive and quantitative method that can be used as an efficient tool to complement individualized treatment.

## Data Availability Statement

The original contributions presented in the study are included in the article/supplementary material. Further inquiries can be directed to the corresponding author.

## Ethics Statement

The studies involving human participants were reviewed and approved by the Ethics Committee of Southern Medical University. Written informed consent for participation was not required for this study in accordance with the national legislation and the institutional requirements.

## Author Contributions

HZ, FY, and GW contributed to conception and design of the study. MC, LY, AQ, WC, and SY organized the database. HZ and FY performed the statistical analysis. HZ wrote the first draft of the manuscript. FY wrote sections of the manuscript. All authors contributed to manuscript revision, read, and approved the submitted version.

## Conflict of Interest

The authors declare that the research was conducted in the absence of any commercial or financial relationships that could be construed as a potential conflict of interest.

## Publisher’s Note

All claims expressed in this article are solely those of the authors and do not necessarily represent those of their affiliated organizations, or those of the publisher, the editors and the reviewers. Any product that may be evaluated in this article, or claim that may be made by its manufacturer, is not guaranteed or endorsed by the publisher.
